# The Adenosine Analogue NITD008 has Potent Antiviral Activity against Human and Animal Caliciviruses

**DOI:** 10.3390/v11060496

**Published:** 2019-05-30

**Authors:** Daniel Enosi Tuipulotu, Tulio M. Fumian, Natalie E. Netzler, Jason M. Mackenzie, Peter A. White

**Affiliations:** 1 School of Biotechnology and Biomolecular Sciences, University of New South Wales, Sydney, NSW 2052, Australia; d.enosi@unsw.edu.au (D.E.T.); tuliomf@yahoo.com.br (T.M.F.); n.netzler@unsw.edu.au (N.E.N.); 2 Laboratório de Virologia Comparada e Ambiental, Instituto Oswaldo Cruz, FIOCRUZ, Rio de Janeiro 21040-900, Brazil; 3 Department of Microbiology and Immunology, Peter Doherty Institute for Infection and Immunity, University of Melbourne, Melbourne, VC 3010, Australia; jason.mackenzie@unimelb.edu.au

**Keywords:** norovirus, antivirals, polymerase inhibitor, nucleoside analogue, caliciviruses

## Abstract

The widespread nature of calicivirus infections globally has a substantial impact on the health and well-being of humans and animals alike. Currently, the only vaccines approved against caliciviruses are for feline and rabbit-specific members of this group, and thus there is a growing effort towards the development of broad-spectrum antivirals for calicivirus infections. In this study, we evaluated the antiviral activity of the adenosine analogue NITD008 *in vitro* using three calicivirus model systems namely; feline calicivirus (FCV), murine norovirus (MNV), and the human norovirus replicon. We show that the nucleoside analogue (NA), NITD008, has limited toxicity and inhibits calicivirus replication in all three model systems with EC_50_ values of 0.94 μM, 0.91 µM, and 0.21 µM for MNV, FCV, and the Norwalk replicon, respectively. NITD008 has a similar level of potency to the most well-studied NA 2′-*C*-methylcytidine *in vitro*. Significantly, we also show that continual NITD008 treatment effectively cleared the Norwalk replicon from cells and treatment with 5 µM NITD008 was sufficient to completely prevent rebound. Given the potency displayed by NITD008 against several caliciviruses, we propose that this compound should be interrogated further to assess its effectiveness *in vivo*. In summary, we have added a potent NA to the current suite of antiviral compounds and provide a NA scaffold that could be further modified for therapeutic use against calicivirus infections.

## 1. Introduction

The *Caliciviridae* family is a group of positive-sense single stranded RNA viruses that are divided into five genera including *Vesivirus*, *Nebovirus*, *Lagovirus, Sapovirus*, and *Norovirus* [1]. Ubiquitous in nature, caliciviruses can infect a wide range of animal hosts, which usually manifest as either upper respiratory infections, acute gastroenteritis (AGE), encephalitis, or haemorrhagic disease [2]. In comparison, calicivirus infections within humans result solely in AGE, which is caused by members of the *Norovirus* and *Sapovirus* genera [3]. Human noroviruses are recognized as a predominant cause of AGE across all ages [4], and are estimated to cause around 700 million infections annually which results in over 200,000 deaths worldwide [5]. Furthermore, human noroviruses have a marked effect on the global economy, costing over $60 billion USD annually, most of which is associated with medical care, hospitalizations and loss of productive days at work [6]. Thus, the pathogenic nature and high prevalence of caliciviruses in human and animals warrants the development of antiviral strategies against this family of viruses.

Currently, there are no approved calicivirus-specific antivirals, and vaccination is only available for a subset of viruses including feline calicivirus (FCV) [7], rabbit haemorrhagic disease virus (RHDV)-1, and RHDV-2 [8], whilst human norovirus vaccines are in Phase II clinical trials [9,10,11,12]. Current animal calicivirus vaccines have biological and logistical shortfalls that limit their efficacy. For example, the RHDV-1 vaccine provides poor cross-protection against RHDV-2, whilst the RHDV-2 vaccine has had limited testing and is currently available only in the UK [8,13,14,15]. Similarly, approved FCV vaccines (including strains F9, 255, 431 and G1) do not provide complete protection against the antigenically distinct virulent systemic FCV (VS-FCV) [7,16,17]. In the absence of efficacious calicivirus vaccines, broad-spectrum antivirals are highly desired to cure or prevent calicivirus infections in animals and humans.

Antiviral compounds screened against caliciviruses so far have mostly involved *in vitro* enzyme and *in vitro* cell based models, notably murine norovirus (MNV), the human GI replicon [18], and to a lesser extent FCV, RHDV, rabbit calicivirus and porcine sapovirus (PSaV) [19,20,21,22,23]. These compounds are broadly divided into host-targeting agents and direct-acting antivirals (DAAs), with the latter targeting virus-encoded proteins essential for infection and replication, including the viral protease and RNA-dependent RNA polymerase (RdRp) [24]. Inhibitors of the RdRp are further divided into nucleoside analogues (NAs) and non-nucleoside inhibitors (NNIs) [2]. NNIs bind to allosteric sites on the RdRp, which prevent conformational changes required for polymerase activity. Alternatively, NAs are incorporated into the newly synthesized viral genome during polymerization, which results in chain termination and inhibition of viral replication. The RdRp is an attractive antiviral target for several reasons: (i) there is no host cell homologue which limits off-target effects; (ii) the highly conserved nature of the active site confers broad-spectrum antiviral activity to NAs; and (iii) many NAs have a high barrier to resistance, which is also related to the conserved nature of the active site where nucleotide incorporation occurs during polymerization [25].

Currently, the antiviral which shows the greatest promise for the treatment of calicivirus infections is 2′-C-methycytidine (2CMC), a NA which was first developed for the treatment of flaviviruses [26], but has since been extensively examined as an antiviral scaffold against caliciviruses *in vitro* and *in vivo* [19,27,28,29,30,31,32]. More recently, the NA 7-deaza-2′-C-methyladenosine (7DMA) has been shown to display low micromolar potency against MNV, PSaV, and the Norwalk replicon [22], although we have previously shown that it has no effect against FCV replication [19] and therefore it does not display broad-spectrum activity against all caliciviruses. For this reason, we chose not to explore this compound in this study. Other NAs have also been screened against members of the *Caliciviridae*, including ribavirin and favipiravir (T-705), yet both displayed poor activity against the human norovirus replicon with half maximal effective concentrations (EC_50_) of 40 μM and 21 μM, respectively [27,30]. Ribavirin and T-705 have also been used clinically in attempt to treat chronic norovirus infections [33,34]. Thus, NAs represent an important therapeutic option for calicivirus infections as they generally display broad-spectrum activity; 2CMC for example has been shown to be effective against several calicivirus model systems including: MNV, FCV, PSaV, human norovirus, the Norwalk replicon [19,22,27,28,29,30,31,32]. 

The adenosine analogue, NITD008, is a promising antiviral compound with a well-defined mode of action, however, has yet to be tested against a calicivirus. NITD008 was originally designed as an antiviral for dengue virus (DENV) [35], but further development was halted due to toxicity in dogs with prolonged treatment [35]. Like many other NAs that have shown to be toxic *in vivo* (e.g., 2CMC and T705), NITD008 has continued to be explored for antiviral efficacy against other viruses in the hope that it can be used as a scaffold for further development into non-toxic derivatives. Since the initial discovery of NITD008, it has also been found to inhibit the replication of several additional flaviviruses including, but not limited to: Zika virus (ZIKV), hepatitis C virus (HCV), West Nile virus (WNV), and Yellow fever virus (YFV) [35,36,37,38]. Moreover, recent studies have shown that NITD008 effectively inhibited replication of enterovirus 71 (EV71) [39,40] of the *Picornaviridae* family and hepatitis E virus of the *Hepeviridae* family [41]. Given the success of NITD008 against the abovementioned viruses we hypothesized that this compound would also inhibit members of the *Caliciviridae* family.

In this study, we evaluated the *in vitro* antiviral effects of NITD008 against a trio of caliciviruses (FCV, MNV and human norovirus) to provide insight on the applicability of this compound and its derivatives as a therapeutic for calicivirus infections. Importantly, we found that NITD008 is as potent as many of the well-published NAs shown to be effective against caliciviruses and has limited toxicity *in vitro*. Moreover, we show that NITD008 displays inhibition against viruses that belong to two genera of the *Caliciviridae* family (*Norovirus* and *Vesivirus*) demonstrating broad-spectrum properties desirable for a calicivirus therapeutic.

## 2. Materials and Methods 

### 2.1. Drugs, Cell Lines, Viruses

2′-C-methylcytidine (2CMC; Sigma-Aldrich, St. Louis, MO, USA) and NITD008 (In Vitro Technologies, VIC, Australia) were dissolved in 100% (v/v) dimethyl sulfoxide (DMSO). CRFK (ATCC CCL-94) cells were maintained in Eagle’s Minimum Medium (EMEM, ATCC 30-2003), whereas RAW264.7 and the Norwalk replicon-bearing Huh7 (HG23) cell lines were maintained in Dulbecco’s modified Eagle’s medium (DMEM, Life Technologies, Carlsbad, CA, USA). All media were supplemented with 10% fetal bovine serum and 100 U/mL penicillin-streptomycin (Life Technologies). HG23 media was further supplemented with 10 mM HEPES and 1.25 mg/mL Geneticin (G418, Life Technologies). FCV (F-9 strain, ATCC VR-782) and MNV-1 (CW1 strain) were used for viral infections. All cell maintenance, virus infections and assays were performed at 37 °C with 5% CO_2_.

### 2.2. Toxicity Analysis

Compound-induced toxicity of mammalian cells was assessed using CellTitre Blue (CTB) reagent (Promega, Madison, WI, USA), as previously described [19,42,43,44,45]. Briefly, CRFK (3.5 × 10^4^ cells/well), RAW264.7 (2 × 10^4^ cells/well) or Huh7 (5 × 10^3^ cells/well) were seeded into 96-well plates and incubated overnight. Monolayers were then treated with increasing concentrations of compound. For consistency, the duration of a toxicity assay was matched to the corresponding antiviral assay. Following incubation, the CTB reagent was added to wells and fluorescence (560_Ex_/590_Em_) was measured as per the manufacturer’s instructions. The half-maximal cytotoxic concentrations (CC_50_) were determined from at least eight concentrations using GraphPad Prism v.7 (La Jolla, CA, USA).

### 2.3. Inhibition of Viral Infectivity

The effect of NITD008 on FCV and MNV infection was assessed by plaque reduction assays, as previously described [19,42,43,44,45]. Here, CRFK (8 × 10^5^) or RAW264.7 (2 × 10^6^) cells were seeded into each well of 6-well plates and incubated overnight for attachment. The following morning, monolayers were infected with approximately 80 plaque-forming units of MNV or FCV. After 1 h, unbound virus was removed and replaced with a semi-solid agarose overlay containing the compound of interest at the desired concentration. Plates were incubated for 24 or 48 h for FCV and MNV, respectively, which is the time required for each virus to generate visible and countable plaques. Plaques were enumerated for each treatment condition and compared the vehicle (0.1% v/v DMSO) control to calculate percentage inhibition. The half-maximal effective concentrations (EC_50_) were determined from at least eight concentrations of antiviral using GraphPad Prism v.7. For drug combination assays, five concentrations of NITD008 (0–1 µM) and 2CMC (0–3 µM) were tested together against MNV in a plaque reduction assay. A dose-response matrix of all combinations was then analysed for synergy or antagonism using the Lowe-Additivity model on SynergyFinder [46,47].

### 2.4. Inhibition of Viral Replication

The effect of NITD008 on replication of FCV, MNV, and the Norwalk replicon was also assessed by measuring viral RNA levels by reverse transcription quantitative PCR (RT-qPCR). For FCV, CRFK cells (2 × 10^5^/well) were infected at a multiplicity of infection (MOI) of 0.0005, whilst for MNV, RAW264.7 cells (5 × 10^5^/cells) were infected at a MOI of 0.01. After 1 h infection, unbound virus was replaced with media containing drug treatment and incubated for 24 h (FCV) or 48 h (MNV). For the Norwalk replicon, HG23 cells (1 × 10^5^/well) were incubated for 24 h in G418-free media before drug treatment for a further 48 h. All experiments were performed in 24-well plates. Following drug treatment, the QIAmp viral RNA kit (Qiagen) and the RNAeasy mini kits (Qiagen) were used for RNA extraction for virus and replicon assays, respectively. RT-qPCR was used to determine viral or replicon RNA levels, as previously described [31,45]. Targets for qRT-PCR, primer sequences, cycling conditions and plasmids for standard curve generation have been previously described [19,31,45].

### 2.5. Clearance-Rebound Studies

Clearance and rebound assays were performed by combining previously described methods [31,48] which are outlined in Figure 4A. These assays provided insight into the ability of NITD008 to inhibit replicon/virus replication to an extent that prevents replication from reoccurring once treatment has completed. For the clearance phase, HG23 monolayers (in T25 flasks) were treated with complete media (without G418) containing either DMSO (vehicle only–0.1% *v*/*v*) or selected concentrations of NITD008 (1, 5 and 10 μM) and passaged every four days over a 12-day period. At each passage, (i) 1.5 × 10^5^ HG23 cells were harvested for RNA extraction and RT-qPCR to quantify Norwalk replicon RNA levels and (ii) 2.5 × 10^5^ HG23 cells were seeded into new T25 flasks with media containing the identical compound concentration for further incubation. 

For the rebound phase, 2.5 × 10^5^ HG23 cells from the second and third passages were seeded into 6-well plates with complete media (containing 1.25 mg/mL G418) in the absence of any drug treatment. After seven days, cells were fixed and stained with 0.5% (w/v) crystal violet for visualization of G418-resistant colonies containing the Norwalk replicon.

## 3. Results

### 3.1. NITD008 is Inhibitory against Animal Calicivirus Infection In vitro

**Figure 1:**
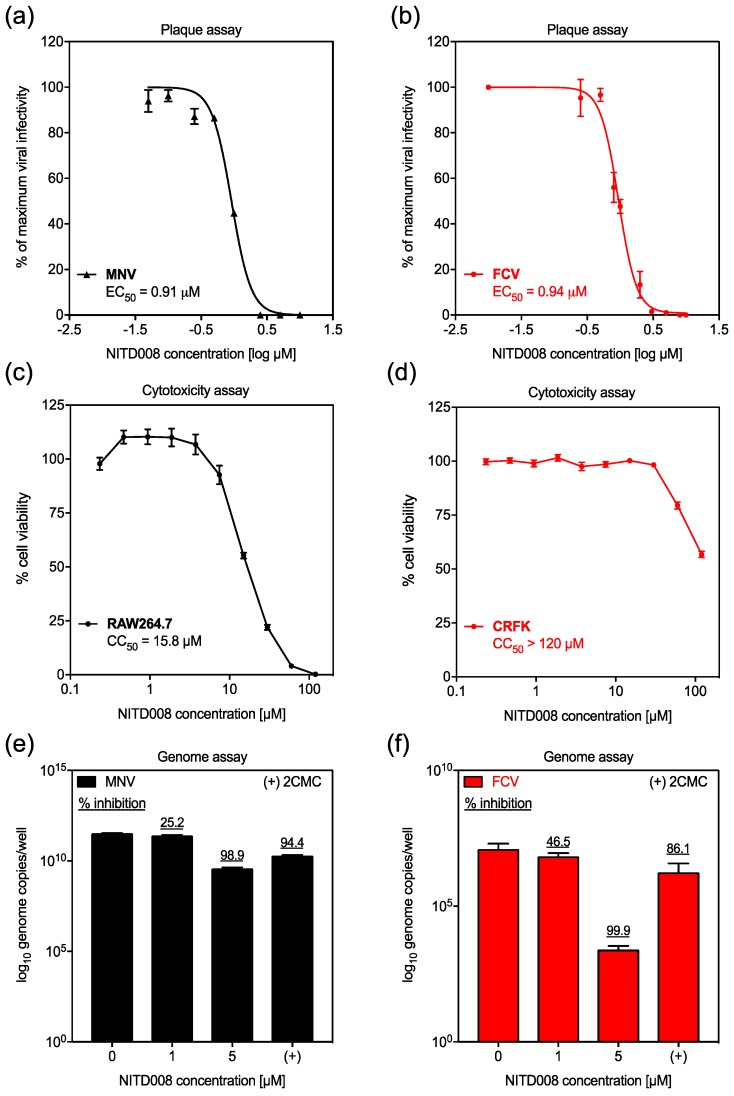
NITD008 is a potent inhibitor of FCV and MNV infection. The antiviral potential of NITD008 was explored against two animal caliciviruses closely related to human norovirus. Panels with black data points/bars are related to MNV and involve 48 h incubations whereas panels with red data points/bars are related to FCV and involve 24 h incubations. (**A**,**B**) Infectious virus levels following NITD008 treatment (0.01–10 μM) were quantified by plaque reduction assay. The percentage of maximal viral infectivity is relative to DMSO controls and is plotted as a dose-response curve. The cytotoxic effects of NITD008 (0.2–120 µM) against (**C**) RAW264.7 and (**D**) CRFK cell lines, permissive for MNV and FCV respectively, were quantified after treatment using the fluorescent CellTiter-Blue assay. The percentage cell viability is relative to DMSO controls. (**E**,**F**) Viral genome levels following NITD008 treatment (1 µM and 5 µM) were quantified by qRT-PCR. The nucleoside analogue 2CMC (10 µM) and DMSO were used as positive and negative controls respectively. Triplicate data from at least two independent experiments are presented for each panel. Error bars represent the mean ± standard deviations.

The antiviral effects of NITD008 were evaluated against FCV and MNV *in vitro* using plaque reduction assays and quantification of viral genomes by RT-qPCR. NITD008 markedly inhibited the replication of both viruses with limited cellular toxicity (Figure 1). Specifically, the EC_50_ of NITD008 was 0.91 µM (95% CI: 0.84–0.99) and 0.94 μM (95% CI: 0.88–1.02) for MNV and FCV, respectively (Figure 1A,B). Furthermore, the CC_50_ value of NITD008 was 15.7 µM (95% CI: 14.23–17.72) for RAW264.7 cells, whereas no toxicity was observed in the CRFK cell line at concentrations up to 120 µM (Figure 1C,D). This was the highest concentration of NITD008 achievable in this assay without causing solvent (DMSO)-induced cellular toxicity. The therapeutic index (TI = CC_50_/EC_50_) for NITD008 was therefore estimated to be 17.2 and ≥127.6 for MNV and FCV, respectively. In addition, we also confirmed the antiviral effects of NITD008 by quantification of viral genomes following treatment at 1 µM and 5 µM. We observed a dose-responsive inhibition of FCV and MNV replication with >90% reduction in genome count at 5 µM (Figure 1E,F). Importantly, we observed that 5 μM NITD008 was more potent than 10 μM 2CMC against both viruses (Figure 1E,F).

### 3.2. NITD008 Combined with 2CMC has an Antagonistic Antiviral Effect

**Figure 2:**
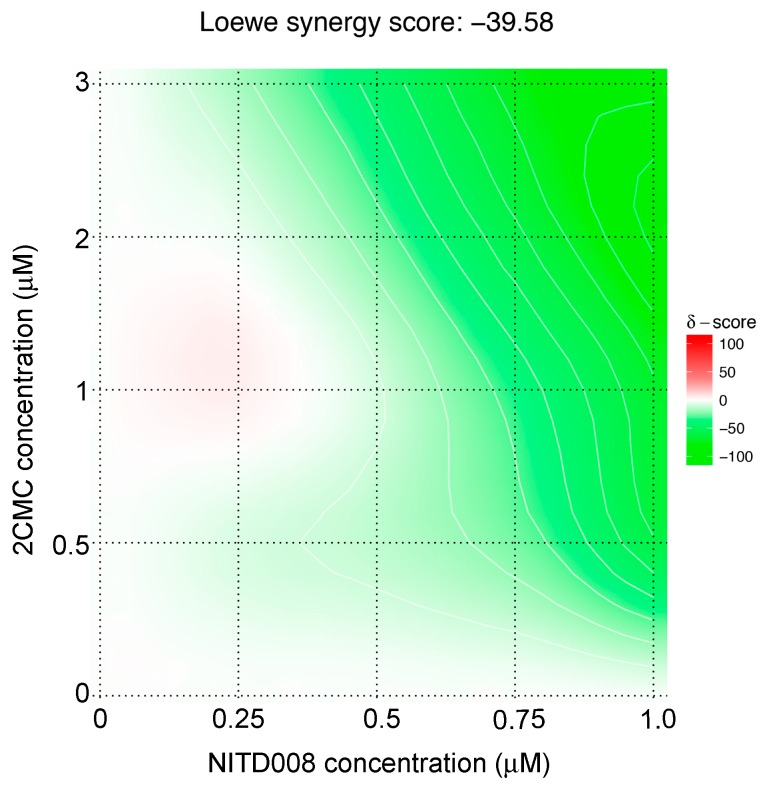
The interaction between the NITD008 and 2CMC is strongly antagonistic. The combinational effects of 2CMC (0–3 µM) and NITD008 (0–1 µM) against MNV infection were tested over a range of concentrations using a plaque reduction assay. The percentage of inhibition observed across all drug combinations were tabled into a dose-response matrix and analyzed for synergy and antagonism using the Lowe-Additivity model on SynergyFinder. The synergy scores obtained for each drug combination across the entire matrix are presented as a heatmap. Synergistic (positive δ-score) and antagonistic (negative δ-score) interactions are represented in red and green respectively, and no observable effect (δ = 0) is represented as white.

NITD008 has been reported to inhibit the replication of numerous flaviviruses and functions as a chain terminator through interaction with the polymerase active site [35,36,37,38,39,40]. To determine whether NITD008 shares the same binding site in caliciviruses, we performed a drug combination analysis with NITD008 and 2CMC (a known calicivirus NA). We tested five concentrations of NITD008 (0–1 μM) and 2CMC (0–3 μM) in combination to assess for synergistic or antagonist effects using a MNV plaque reduction assay. The Lowe-Additivity model demonstrated a strong antagonism between the two compounds (average delta synergy score −39.58 across the matrix), which was more prominent with increasing drug concentrations (delta synergy score–76.79 at the combined maximum drug concentration (Figure 2). 

### 3.3. NITD008 is a Potent Inhibitor of Human Norovirus Replication

**Figure 3:**
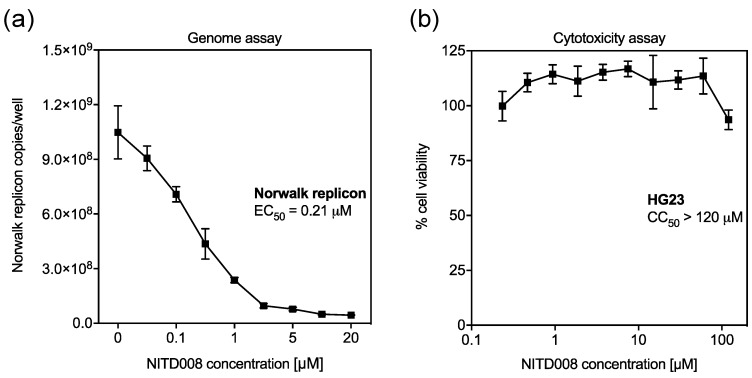
NITD008 is a potent inhibitor of human norovirus replication. (**A**) Norwalk replicon levels were measured using RT-qPCR following NITD008 treatment (0–20 µM) for 48 h. The norovirus RdRp sequence was amplified for the replicon and *β-actin* was amplified as the normalizer for the ΔΔ*C_T_* method (where *C_T_* is threshold cycle). (**B**) The cytotoxic effects of NITD008 (0.2–120 µM) against the hepatoma cell line (Huh7) were assessed after 48 h using the fluorescent CellTitre-Blue assay. The percentage cell viability is relative to DMSO controls. Triplicate data from two independent experiments are presented for each panel. Error bars represent the mean ± standard deviations.

To determine if NITD008 has antiviral activity against human norovirus, we examined its inhibitory effects against the Norwalk virus replicon using RT-qPCR to measure RNA levels (Figure 3). NITD008 effectively reduced Norwalk replicon levels in a dose-dependent manner (EC_50_ of 0.21 µM, 95% CI: 0.17–0.26) (Figure 3A) and displayed no toxicity against the HG23 cell line when tested up to 120 µM (Figure 3B). The TI for NITD008 in this replicon system was calculated at ≥571.4.

### 3.4. NITD008 Effectively Clears the Human Norovirus Replicon from Cells

**Figure 4:**
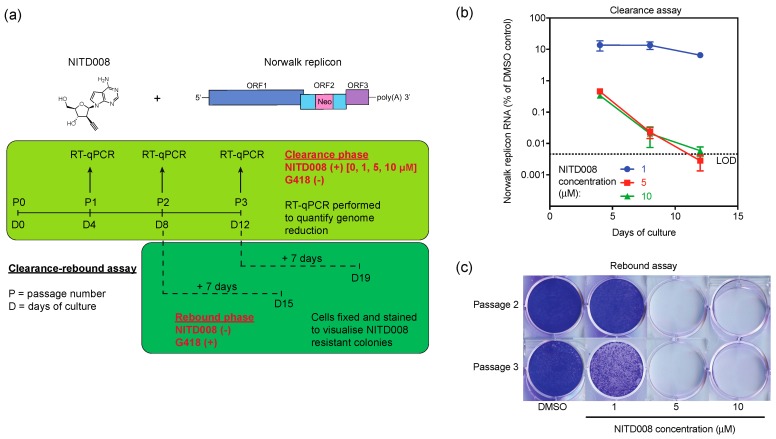
NITD008 effectively clears the Norwalk replicon from replicon-bearing cells. (**A**) Schematic overview of the clearance-rebound assays performed against the Norwalk replicon. The light green panel represents the clearance phase in which replicon-bearing cells were passaged every 4 days in the presence of DMSO or NITD008 at 1, 5, and 10 µM (~ 5, 25, 50× the EC_50_ value). The dark green panel represents the rebound phase in which NITD008 is removed from the media and G418 is used to selected for replicon-bearing cells. (**B**) Clearance of the Norwalk replicon from cells was determined by RT-qPCR. Norwalk replicon levels following NITD008 treatment at every passage are plotted relative to the corresponding DMSO controls. The dotted line represents the qPCR limit of detection. (**C**) Rebound of the Norwalk replicon was assessed by performing colony formation assays on cells passaged in the presence of NITD008 for either 8 or 12 days. Colonies were fixed and stained with crystal violet before visualization. Triplicate data from at least two independent experiments are presented for each panel. Error bars represent the mean ± standard deviations.

To evaluate whether repeated NITD008 treatment could eliminate the Norwalk replicon from host cells, clearance-rebound assays were performed, as described in Figure 4A. During the clearance phase, G418 was absent from the culture media and HG23 replicon-bearing cells were passaged in the presence of either 1, 5, or 10 µM of NITD008 for 12 consecutive days. Norwalk replicon RNA levels were measured by RT-qPCR at each passage for all concentrations of NITD008 tested and compared to the mock control (Figure 4A). Following the first passage treatment with 1 µM, NITD008 resulted in an 86.3% ± 4.9% reduction in replicon RNA levels, whereas treatment with 5 µM and 10 µM resulted in a 99.5% ± 0.1% and 99.7% ± 0.1% reduction, respectively (Figure 4B). At the third passage, 1 µM reduced the replicon population by 93.4% ± 0.8% and treatment at 5 and 10 µM reduced the population by more than 99.9% (Figure 4B).

To determine whether the genome reduction observed in the clearance phase was sufficient to prevent a resurgence of replicon replication, a colony formation assay was used to check for rebound (Figure 4C). Cells from the second and third passage (P2 and P3) at each drug concentration of the clearance-phase were incubated in the presence of G418 to select for replicon-bearing cells (Figure 4A). We observed no rebound for NITD008 treatment at 5 µM and 10 µM at either P2 or P3 (Figure 4C), however, there was complete rebound with 1 µM treatment at P2 and partial rebound at P3 (Figure 1C). In summary, the effectiveness of both clearance and prevention of replicon rebound was enhanced by increasing both NITD008 dosage and treatment duration.

## 4. Discussion

In the absence of efficacious calicivirus vaccines, broad-spectrum antivirals represent a practical solution to limit calicivirus disease and transmission. NAs are a class of antiviral drug that generally display broad-spectrum antiviral activity, which is attributed to the highly conserved polymerase active site that they interact with. So far, the only NAs shown to be effective against caliciviruses include 7DMA, T-705, ribavirin, 2CMC and its derivatives [22,33]. Of these, 2CMC is the most effective and has low micromolar potency against caliciviruses, with reported EC_50_ values of 2.6 µM, 1.6 µM, and 1.3 µM against FCV, MNV, and the Norwalk replicon, respectively [19,30,32]. In this study, we report that NITD008 has EC_50_ values of 0.94 µM, 0.91 µM, and 0.21 µM against FCV, MNV, and the Norwalk replicon systems, respectively. The high level of potency displayed by NITD008 across all three models, and the comparable level of *in vitro* efficiency between NITD008 and previously published NAs, demonstrates that NITD008 is an excellent candidate for further studies and eventual refinement as an antiviral agent for calicivirus infections.

The potency displayed by NITD008 against caliciviruses is comparable to that against other viruses including ZIKV, DENV, and EV71 with EC_50_ values of 0.24 μM, 0.64 μM, and 0.67 μM, respectively [35,36,40]. Despite this potency, NITD008 has not yet progressed to clinical trials for the treatment of virus infections. Pre-clinical animal testing of NITD008 for DENV infection revealed toxicity in dogs following two weeks of daily compound treatment [35]. However, *in vivo* toxicity was absent within the first week of this study and thus derivatives NITD008 may still be a viable treatment of virus infections with a short treatment schedule or instead could be used as a scaffold for further pharmacological improvement. Indeed, we observed toxicity against the RAW264.7 cells with a CC_50_ of 15.8 µM, yet this value is >15-fold higher than the EC_50_ and confirmed that the antiviral activity observed is not a result of cell death.

To confirm that NITD008 inhibited calicivirus replication through interaction with the active site, we tested the combined effects of NITD008 and 2CMC against MNV infection. We show that when combined, a strong antagonistic effect was observed. This antagonism is most likely a result of competition for the active site within the polymerase and demonstrates that NITD008 likely works by a typical NA mechanism. The antiviral activity displayed by NITD008 against members of the *Caliciviridae* (cross-genera and cross-genogroup) supports the idea that it could be used widely for the treatment of animal and human calicivirus infections including variants that emerge through rapid evolution.

Next, we wanted to assess whether NITD008 could effectively prevent viral rebound after completion of a short treatment regime. We exposed the Norwalk replicon to NITD008 for 12 days and quantified replicon RNA at multiple time points to determine whether treatment could reduce replication and subsequently prevent replicon rebound. A decrease in the replicon RNA levels was observed with consecutive passages in the presence of NITD008 (between 1 μM and 5 μM, and between 5 μM and 10 μM) and treatment with 5 µM for four days was sufficient to completely prevent rebound of the Norwalk replicon. The ability of NITD008 to prevent viral rebound is likely to be a critical factor towards the therapeutic efficacy of this compound against caliciviruses. However, these experiments were performed *in vitro* and thus the concentrations of NITD008 used in this study may not reflect clinically effective dosages. Nonetheless, our preliminary findings warrant more extensive assessment of NITD008 using *in vivo* studies. This would involve screening NITD008 against MNV using the well described mouse model infection [31] to determine whether treatment can reduce MNV-induced diarrhoea, reduce viral load, prevent mortality and limit viral transmission.

The development of antiviral resistance is important clinically since rebound of resistant populations can have a dramatic impact on treatment success [49]. Several NITD008 resistance mutations have been documented in EV71 [40] and HCV [37] following passage of these viruses with increasing concentrations of NITD008. For EV71, mutations that confer resistance are within the 3A and 3D domains which encode replication complex proteins, including viral the polymerase [40]. For HCV, mutations occur within motif B of the polymerase, as well in the finger and thumb domains [37]. In comparison, other studies have shown that long-term passage in the presence of NITD008 failed to induce mutations within DENV [35], WNV [35], ZIKV [36], and EV71 [39]. For the two studies on EV71, which show conflicting data (i.e., resistance induced versus no resistance induced) in response to continual NITD008 treatment [39,40], this is likely attributed to the different experimental techniques performed for resistance selection. However, it is clear from these studies that resistance only emerged with long-term exposure and overall NITD008 has a high barrier to resistance. Although we show that the cell line was completely cured of the Norwalk replicon with NITD008 treatment, we did not perform an analysis to detect antiviral escape mutants. Future evaluation of resistance to NITD008 within caliciviruses should be performed to better characterise the clinical applicability of this compound.

In summary, we present a new potent NA to the current suite of compounds with antiviral activity against caliciviruses. The sub-micromolar potency, low toxicity, large therapeutic window of inhibition displayed by NITD008 against several caliciviruses *in vitro* suggest that this compound will be of considerable benefit as a scaffold for therapeutic development. Moreover, since resistance profiles against NITD008 have been documented, combination therapy with other DAAs would be an appropriate strategy to limit antiviral resistance to NITD008. Our data provide evidence that NITD008 would likely be a suitable backbone for calicivirus therapies and further investigations into potential drug combinations are warranted.
